# Disentangling the External Reference Frames Relevant to Tactile Localization

**DOI:** 10.1371/journal.pone.0158829

**Published:** 2016-07-08

**Authors:** Tobias Heed, Jenny Backhaus, Brigitte Röder, Stephanie Badde

**Affiliations:** 1 Biological Psychology and Neuropsychology, Faculty of Psychology and Human Movement Science, University of Hamburg, Hamburg, Germany; 2 Department of Internal Medicine 1, UKSH Campus Lübeck, Lübeck, Germany; 3 Department of Psychology, New York University, New York, New York, United States of America; Ecole Polytechnique Federale de Lausanne, SWITZERLAND

## Abstract

Different reference frames appear to be relevant for tactile spatial coding. When participants give temporal order judgments (TOJ) of two tactile stimuli, one on each hand, performance declines when the hands are crossed. This effect is attributed to a conflict between anatomical and external location codes: hand crossing places the anatomically right hand into the left side of external space. However, hand crossing alone does not specify the anchor of the external reference frame, such as gaze, trunk, or the stimulated limb. Experiments that used explicit localization responses, such as pointing to tactile stimuli rather than crossing manipulations, have consistently implicated gaze-centered coding for touch. To test whether crossing effects can be explained by gaze-centered coding alone, participants made TOJ while the position of the hands was manipulated relative to gaze and trunk. The two hands either lay on different sides of space relative to gaze or trunk, or they both lay on one side of the respective space. In the latter posture, one hand was on its "regular side of space" despite hand crossing, thus reducing overall conflict between anatomical and external codes. TOJ crossing effects were significantly reduced when the hands were both located on the same side of space relative to gaze, indicating gaze-centered coding. Evidence for trunk-centered coding was tentative, with an effect in reaction time but not in accuracy. These results link paradigms that use explicit localization and TOJ, and corroborate the relevance of gaze-related coding for touch. Yet, gaze and trunk-centered coding did not account for the total size of crossing effects, suggesting that tactile localization relies on additional, possibly limb-centered, reference frames. Thus, tactile location appears to be estimated by integrating multiple anatomical and external reference frames.

## Introduction

Many behavioral, neurophysiological, and electrophysiological studies have suggested that the brain transforms spatial information from all senses into a gaze-centered reference frame. The term gaze refers to the line of sight, that is, where the eyes are directed as a consequence of the combination of head position and the position of the eyes in the head. Whereas the relevance of gaze position is immediately apparent for vision, it is less obvious for other modalities. Nevertheless, human participants systematically mislocalize the location of tactile stimuli in the direction of gaze when they indicate tactile stimulus location by referring to a ruler [1], as well as when they point to the tactile location [2,3], and these errors are similar to misestimates of eye position in the dark [4]. Similar gaze-dependent error patterns have been observed for visual, auditory, and proprioceptive reach targets [3,5,6]. Magnetoencephalographic (MEG) recordings provide neural evidence for the relevance of gaze in tactile spatial coding. When human participants planned saccades or pointing movements towards tactile target stimuli, parietal oscillatory brain activity in the alpha frequency range (8–12 Hz) was lateralized relative to gaze [7,8]. Finally, gaze position can even be relevant for such high-level tasks as tactile object recognition; for instance, directing gaze towards the unseen tactile object sometimes accelerated its identification [9].

Whereas these experiments have explicitly tested for the relevance of a gaze-centered reference frame, another line of experiments has used temporal order judgments (TOJ) in combination with limb crossing to investigate tactile reference frames more generally. In the TOJ task participants judge which of two tactile stimuli, each applied to a different limb, occurred first. Participants perform significantly worse with crossed than with uncrossed limbs [10–15]. With crossed hands, anatomical, that is, skin-based, and external reference frames point in different directions: for instance, the right hand is then located in left external space. This conflict probably causes the TOJ crossing effect. Note, that the term "external", that is frequently used in the tactile localization literature, does not refer to a reference frame that is independent of the observer; rather, the term denotes that a transformation from the original skin space has occurred, and that posture has been taken into account to localize the stimulus in space (see [16,17]). However, the anchor of the external reference frame is unspecified because hand crossing reverses the left-right dimension relative to many potentially relevant anchors, such as the eyes, the head, and the trunk, in a typical experimental setup in which eyes, head, body, and arms all face straight [16].

Experiments that create reference frame conflict using crossed postures imply that tactile localization uses an external reference frame. This assertion fits well with the body of studies demonstrating the relevance of gaze for tactile spatial coding. Nevertheless, there is currently only indirect evidence that this external reference frame is, indeed, a gaze-centered one. Congenitally blind individuals have either shown no TOJ crossing effects [18], or only small effects that markedly differed from those of sighted individuals [19]. Consistently, electrophysiological activity is modulated by an external reference frame in sighted, but not in congenitally blind participants [20,21]. These findings suggest a pivotal role of the visual system for tactile spatial coding, and may therefore imply that the external reference frame in TOJ tasks is gaze-centered.

However, other experimental results, obtained by modifying crossed postures in tactile localization paradigms, challenge the notion of merely a gaze-centered reference frame. Patients suffering from hemispatial neglect often cannot report the occurrence of tactile stimulation to the hand that is anatomically contralateral to the lesion [22,23]; in the related phenomenon of extinction, a contralesional stimulus goes undetected when an ipsilesional stimulus is presented concurrently [23,24]. These deficits were ameliorated when patients crossed their hands [23], presumably because the tactile stimulus now occurred in the external space of the non-affected hemifield. Yet, when patients placed both hands in the affected or both in the unaffected hemifield, the spatially right stimulus was still detected more often than the left stimulus [24]. This finding speaks against the relevance of a gaze-centered reference frame. Instead, extinction appears to have operated based on the relative position of the two hands. A TOJ study in healthy participants supported this view: In one condition of this study, the right hand was placed in the opposite hemifield, and the left arm was placed around it, so that each hand came to lie in the opposite hemifield, but the arms were not crossed (see Figure 5D in [10]). Note that, in this latter posture, the right hand is to the left of the left hand's midline, with the midline defined as the line that continues from the arm, through the hand and the middle finger. Thus, for the left hand, its placement is in conflict with anatomy, just like in the normal arms-crossed posture. In contrast, the left hand is to the left of the right hand's midline, that is, the relative locations of the two hands are in accord with anatomy. Therefore, the crossing effect should be reduced in this arms-not-crossed posture if a hand-centered reference frame were relevant for tactile spatial coding. Indeed, the crossing effect was reported to be much smaller in the arms-not-crossed condition [10].

Reliance on a hand-centered, rather than a gaze-centered, reference frame in tactile TOJ tasks seems to be at odds with the many findings implying that tactile localization uses gaze-centered spatial coding. However, the body of experimental findings with the TOJ paradigm and other hand crossing paradigms has suggested that tactile localization integrates and weights different kinds of reference frames, even if this processing principle has so far always been demonstrated for anatomical and (not further specified) external reference frames. It is therefore possible that the brain uses several external reference frames in parallel for tactile localization, rather than just a single, hand- or gaze-centered one. All of these different reference frames would be integrated to obtain a maximally precise tactile location estimate [14,16].

The aim of the present study was two-fold. First, to better link limb crossing studies with studies that investigated errors in explicit location judgments, such as pointing tasks, we tested whether a gaze-centered reference frame affects the posture effect in TOJ as well. Second, to test the idea that not a single, but several external reference frames are integrated for tactile spatial coding, we additionally investigated influences of a trunk-centered reference frame in the TOJ task.

### Experimental rationale

Based on the idea that humans integrate multiple kinds of spatial information to arrive at optimal tactile localization decisions, we reasoned that TOJs should be affected by whether the two hands are placed in separate sides of space relative to different reference frames.

For any reference frame, TOJs with crossed hands may be easier when the two hands reside on one common side rather than on opposite sides. This is because the different sides, relative to the reference frame's anchor or midline, may be salient spatial markers that are critical for spatial conflict to emerge. To illustrate, assume that tactile localization were affected by a gaze-centered reference frame. When gaze is directed across the midline between the two crossed hands, then the hands reside in the visual hemifield that is opposite to the body side to which they belong (the right hand in the left hemifield and vice versa). In contrast, when gaze is directed such that the hands are located in the same hemifield, then anatomical and external locations are opposed for one, but not for the other hand. For instance, when gaze is straight ahead, but the hands are positioned to the right of the body, then the right hand is located in the right hemifield even when the hands are crossed. Therefore, anatomical and external information are not in conflict for this hand, and, thus, the overall amount of conflict is reduced. An analogous argument can be made for a trunk-centered reference frame. Accordingly, performance with crossed hands should improve when both arms are positioned to one side relative to a given reference frame than when they are positioned straight ahead.

Whereas modulations of performance in crossed postures are well established, previous reports on modulations of TOJ performance with uncrossed postures have been mixed. Some studies did [25–27], whereas other studies did not [13,28,29] observe a modulation of TOJ effects with uncrossed hands. Therefore, TOJs with uncrossed hands may not be sensitive to different external reference frames in the current study, for instance due to ceiling effects. However, if performance in uncrossed postures were sensitive to the manipulation of reference frames, then the result pattern should be different from that with crossed hands. The different sides of stimulation, relative to a given reference frame, may serve as information to individuate the stimuli, in consequence making it easier to discriminate them. This reasoning is supported by the finding that TOJs between a visual and a tactile stimulus were performed more successfully when the two stimuli were presented on different, rather than in the same, side of space [30,31]. Thus, if tactile localization were affected by a gaze-centered reference frame, then it should be easier for participants to make TOJ when the eyes are directed such that each hand lies in its own visual hemifield, than when both hands lie in the same visual hemifield. An analogous argument can be made for a trunk-centered reference frame. Accordingly, TOJ performance with uncrossed hands may be worse when the hands lie in the same part of space with respect to a reference frame that is relevant to tactile localization.

Viewed together, positioning the hands on one side with respect to a given reference frame should lead to improved TOJ performance with crossed hands. At the same time, positioning uncrossed hands on one side may lead to impaired TOJ performance with uncrossed hands, though null results for uncrossed postures in previous studies suggest that such an effect may well be absent. These predictions consistently lead us to expect a reduction of the hand crossing effect, defined as the difference between uncrossed and crossed TOJ performance, for postures in which the hands lie on the same side relative to a given reference frame.

## Experiment 1

### Participants

Participants were recruited from the student body of the University of Hamburg and received course credit or monetary compensation (6 € / hr) for participation. Twenty-four participants, 8 of them female, took part in Experiment 1, aged 20–39 years (mean: 26 years). All were right-handed, had normal or corrected-to-normal vision, and did not report any tactile deficits and neurological disorders. The experiments in this paper were conducted in accordance with the guidelines of the German Association of Psychology and with the guidelines laid down in the Declaration of Helsinki. Participants gave their verbal consent. They were told that they could stop the experiment at any time, and were asked whether they wanted to participate after they had read the instructions, which also stated, in written form, the possibility to leave at any time. All data, including personal attributes such as age and visual status, were stored only with reference to a running number, not to participants' names.

### Experimental Design

**Fig 1:**
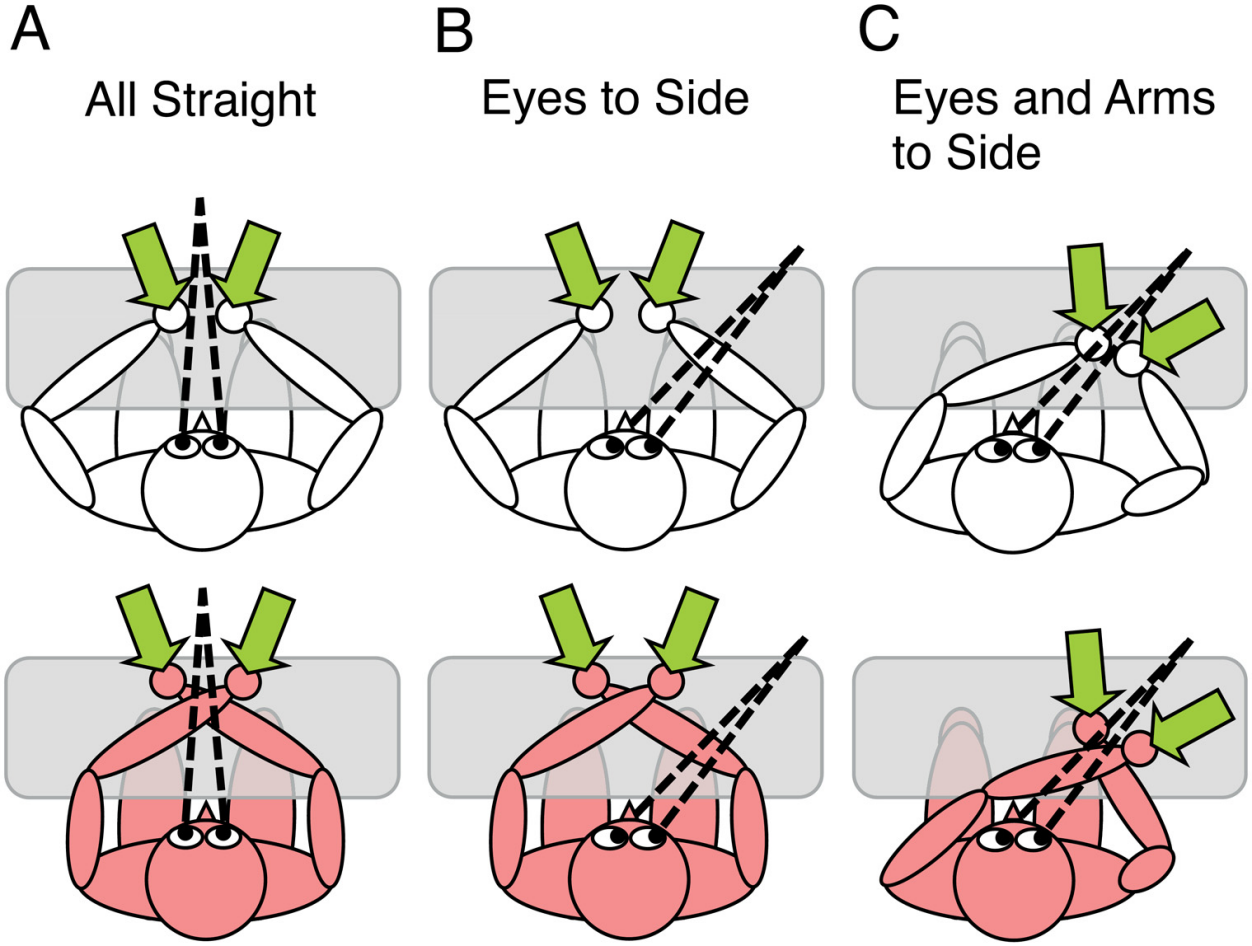
Experimental conditions of Experiment 1. (A-C) Posture conditions. Upper vs. lower row: uncrossed vs. crossed hand conditions. Dotted lines indicate fixation. Green arrows depict the locations of the two tactile stimuli for the temporal order judgment. See text for details.

Experiment 1 compared the relevance of a gaze-centered with a trunk-centered reference frame for tactile localization. We used three combinations of eye and arm position, referred to as Posture Conditions from hereon, to manipulate the relative positions of the arms relative to gaze and trunk (see Fig 1).

The baseline condition was *All Straight*, with arms, head, and eyes all facing straight (Fig 1A).

In condition *Eyes To Side*, arms and head faced straight, but the eyes were directed to the right side (Fig 1B), so that arms and gaze were misaligned. Whereas in *All Straight*, the arms were located in opposite sides of space relative to gaze, in *Eyes To Side* they were located in one side of space relative to gaze. In contrast, arm position was aligned with the trunk midline in both conditions. Thus, *All Straight* and *Eyes To Side* differed only with respect to gaze, and all other postural parameters remained identical between them.

In *Eyes And Arms To Side*, the arms were brought to the right of the head and body, so that the arms were now aligned with gaze, but not aligned with the trunk midline (Fig 1C).

Thus, although *Eyes To Side* and *Eyes And Arms To Side* differed only in arm position, with all other postural parameters identical between them (Fig 1B vs. 1C), they manipulated arm posture in opposite ways relative to gaze and trunk. If tactile localization depended exclusively on a trunk-centered reference frame, the TOJ crossing effect should be reduced relative to *All Straight* in *Eyes And Arms To Side*, but not in *Eyes To Side* (Fig 1, crossing effect 1C < 1A, 1B = = 1A). Similarly, if tactile localization exclusively depended on a gaze-centered reference frame, then the TOJ crossing effect should be reduced relative to *All Straight* in *Eyes To Side*, but not in *Eyes And Arms To Side*, (Fig 1, crossing effect 1B < 1A, 1C = = 1A). Finally, if both types of reference frames were relevant, then both *Eyes To Side* and *Eyes And Arms To Side* should reduce the TOJ crossing effect with respect to *All Straight* (Fig 1, crossing effect 1C < 1A, 1B < 1A).

In each condition, TOJ were administered with uncrossed and crossed hands, resulting in 6 conditions (3 head/eye/arm postures x uncrossed/crossed hands).

Stimuli were presented with a stimulus onset asynchrony (SOA) of -1000, -700, -400, -200, -150, -110, -80, -50, -20, 20, 50, 80, 110, 150, 200, 400, 700, and 1000 ms, with negative SOAs denoting that the left stimulus was presented first. Each combination of condition and SOA was presented 20 times. Experiments were divided into blocks of 90 trials, and participants were encouraged to take breaks between blocks. Posture Conditions and Crossing Status were varied blockwise in pseudo-randomized order. SOA was varied pseudo-randomly within blocks. Data acquisition for each experiment took approximately three hours and was divided into two sessions of 90 minutes each, separated by a maximum of one week.

### Materials

Participants sat on a comfortable chair. The hands were placed 30 cm apart in both uncrossed and crossed conditions. Responses were given by lifting the index finger from a custom-built light gate that was embedded into a small plastic cube with an indentation for finger placement. The experiment was controlled by the software Presentation (Neurobehavioral Systems, Albany, CA, USA), version 11.0, which interfaced with custom-built hardware to drive stimulators and record responses.

Stimuli were 15 ms long vibrations of 167 Hz, delivered through Oticon bone conductors (Oticon Ltd., Milton Keynes, UK, type BC 461–012, sized about 1.6 x 1.0 x 0.8 cm) attached to the first and second phalanx of the index fingers. Participants wore ear plugs and heard white noise played through headphones to mask any noise made by the tactile stimulators.

Participants fixated one of two fixation points on a 22'' computer screen. The fixation points were positioned on the far left and right of the monitor. The monitor was placed slightly off-center to the right, so that participants had to direct their gaze straight ahead to fixate the left fixation dot, and to deviate gaze about 35° to the right to fixate the right fixation point. Only the currently relevant fixation point was shown in any given trial. Participants placed their head onto a chin rest. Eye position towards fixation was controlled by an eye tracker (EyeLink 1000, SR Research, Kanata, Ontario, Canada), sampled at 500 Hz.

### Procedure

For each stimulus pair, participants responded which stimulus had occurred first by lifting the respective finger. Thus, no translation into a verbal code ("left", "right") was necessary for response coding. A response was acknowledged by a 100 ms long 1000 Hz tone, independent of whether the response was correct. If participants responded before the second stimulus had occurred, this was indicated with a sequence of three 100 ms tones (1000, 900, 1000 Hz), separated by a 100 ms gap. If no response was registered until 2000 ms after the second stimulus, this was indicated by three 100 ms long 1000 Hz tones. Trials ended with the participant's response. The response was followed by a variable interval of 500–800 ms; after this interval, fixation had to remain on the fixation point for 500 ms for the next trial to start. Trials were repeated if fixation had been broken during the trial.

### Statistical analysis

Statistical analysis was performed in R [32] and the R package lme4 [33]. Data figures were produced using the package ggplot2 [34].

We excluded trials from the analysis if RT was lower than 100 ms or higher than two standard deviations from the mean of the respective trial's condition (4.8% of all trials). Because error probability is very high in TOJ tasks, RT was analyzed for both correct and incorrect trials.

We used linear mixed modeling (LMM) to analyze RT, and generalized linear mixed modeling (GLMM) with a logistic link function to analyze accuracy [19]. Log-link GLMMs account for non-linear characteristics of percentage data and for repeated measurements [35]. Random intercepts and slopes per participant were estimated for each main effect to reduce the influence of between-subjects variance on fixed effects estimation [36].

It has been shown that conclusions about TOJ crossing effects are usually comparable when analysis does or does not account for SOA [13,28]. Here, we display psychophysical curves with data points for each SOA to allow comparison to previous studies as well as assessment of potential ceiling effects, but collapsed over SOAs for analysis for several reasons. First, the crossing effect is usually evident in short and long SOAs. Therefore, modeling performance across SOA involves fitting a sigmoid function with variable asymptotes. However, psychophysical curves in the crossed condition often do not follow a sigmoidal pattern, but instead show a reversed slope at short SOAs, resulting in an N-shaped response curve [10]; Both variable asymptotes and N-shape cannot currently be modeled in the context of a GLMM contrast. Second, models that account for the N-shape and the differences in asymptotes [10] rely on different numbers of parameters in uncrossed and crossed conditions, implying that additional processes are at work in the crossed posture. This assumption is, however, debated. Furthermore, we have found the fitted parameters of this model to be less sensitive than other measures [13]. Third, many previous studies have assessed the slope of probit-converted response curves. However, this approach implies ignoring data of longer SOAs, usually beyond SOAs of 110 ms, that is, more than half of the current study's data. Finally, collapsing over SOAs reduced computational and model complexity.

We modeled Crossing Status (uncrossed vs. crossed hands), as well as Posture Condition (2) and Posture Condition (3) as predictors. Posture Condition (1), in which arms, trunk, and gaze were all aligned, served as base level for Posture Conditions (2) and (3).

We used treatment coding (also known as dummy coding) as statistical contrast for all factors. Consequently, simple effects must be interpreted with respect to the base levels of the other factors, whereas the highest interactions relate to both levels of a factor. We devised separate models with uncrossed and crossed hands as base level for Crossing Status to separately assess effects of the Posture Conditions on TOJ performance with uncrossed and crossed hands. Using this releveling procedure rather than post-hoc testing allows estimating parameters within models while accounting for the entire dataset, rather than just a data subset. Thus, variance that is due to high-level interactions is partitioned out from variance that is due to lower level effects [37]. To compensate for deviations from the normal distribution, RT was Box-Cox transformed with lambda = -0.22.

We estimated model parameters with LaPlace approximation, as implemented in the lme4 package. For each model, we conducted 10,000 parametric bootstrap runs to approximate reliable 95% confidence intervals as well as p-values indicating whether parameter estimates significantly differed from zero.

### Results

**Fig 2:**
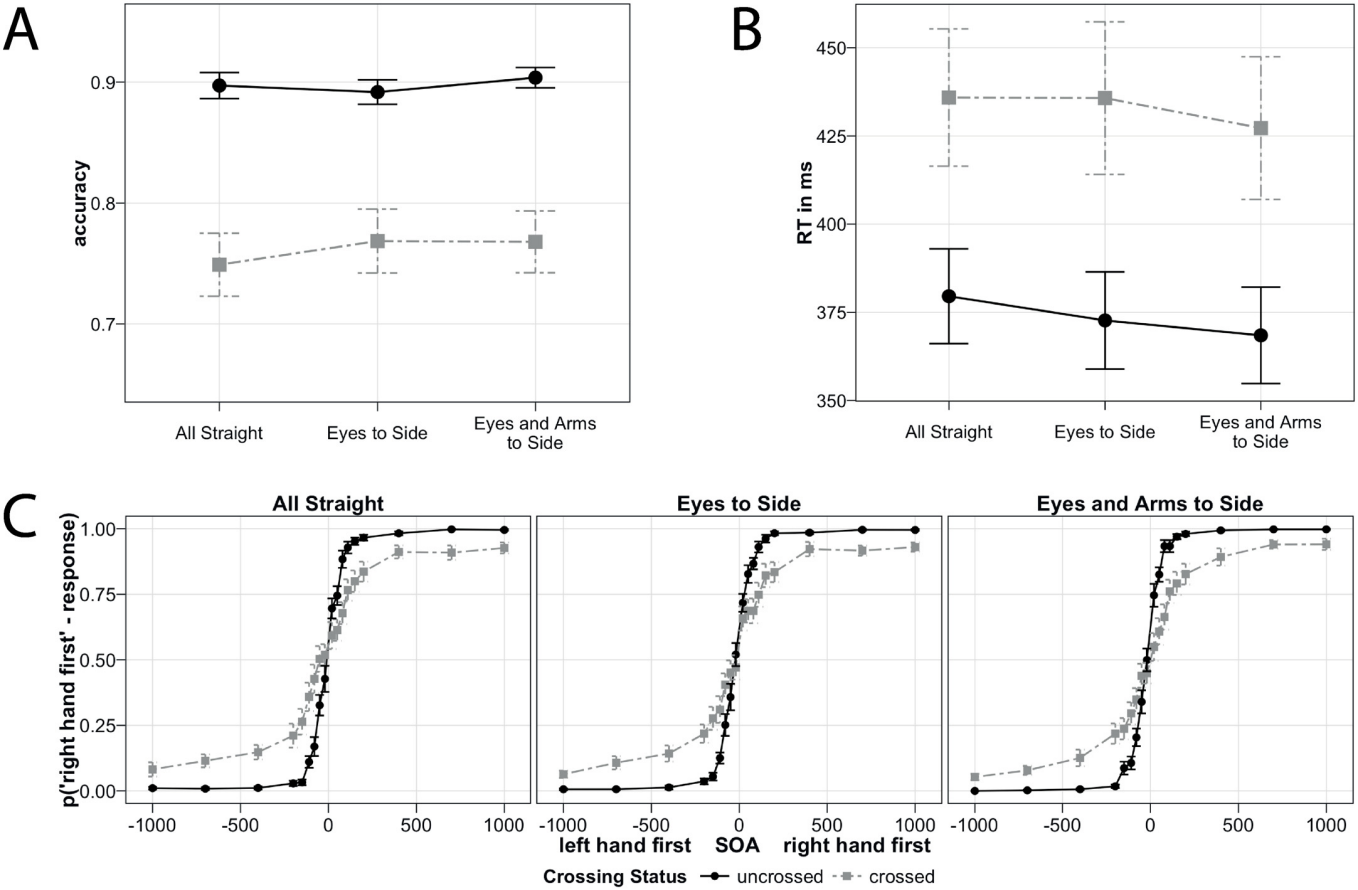
Results of Experiment 1. (A) Accuracy, depicted as the probability to report the correct stimulus, pooled across SOA. (B) Reaction time. (C) Accuracy recoded into proportions of ‘right hand first’ responses by SOA. (A,B,C) Black vs. grey color: uncrossed vs. crossed hand conditions. Error bars depict standard errors of the mean. Note, that y-axes do not start at 0.

Accuracy. Accuracy results are presented pooled across SOA in Fig 2A and recoded into ‘right hand first’- responses by SOA in Fig 2C. Crossing Status significantly affected TOJ performance (β = 1.06, 95% confidence interval for this parameter (0.80, 1.32), p < 0.001, assessed by parametric bootstrapping). Crossing Status interacted with *Eyes To Side* (β = -0.18 (-0.29, -0.07), p = 0.007), indicating that the crossing effect, that is, the difference between uncrossed and crossed hands, was modulated by whether gaze was aligned with the hands, that is, whether both hands were located in one or in separate visual hemifields. In the model that used crossed hands as baseline, TOJ performance improved when the hands were located in one rather than in two visual hemifields (β = 0.12 (0.03, 0.21), p = 0.024). In the releveled model that used uncrossed hands as baseline, TOJ performance did not significantly differ between *Eyes To Side* and *All Straight* (β = -0.06 (-0.16, 0.5), p = 0.365). Thus, the crossing effect was significantly reduced in *Eyes To Side*, driven by accuracy improvement in the crossed, but not the uncrossed posture.

In contrast, there was neither an interaction of Crossing Status and *Eyes And Arms To Side* (β = -0.04 (-0.15, 0.07), p = 0.574), nor were there significant main effects of uncrossed (β = 0.05 (-0.08, 0.17), p = 0.518) and crossed hands (β = 0.09 (-0.02, 0.20), p = 0.184) on TOJ accuracy in *Eyes And Arms To Side*.

To compare *Eyes To Side* and *Eyes And Arms To Side* directly, we releveled the GLMM with *Eyes To Side* as baseline condition. There was an interaction between Crossing Status and *Eyes and Arms to Side* (β = 0.14 (0.03, 0.25), p = 0.038), indicating that the TOJ crossing effect was different between *Eyes to side* and *Eyes and Arms to Side*. However, the simple effects, comparing *Eyes and Arms to* Side, as well as the baseline, to *Eyes to side* separately for each crossing status, were not significant for uncrossed (β = -0.03 (-0.14, 0.07), p = 0.605) or crossed hands (β = 0.1 (-0.02, 0.23), p = 0.159).

In sum, the crossing effect in accuracy appears to have varied with alignment of the hands with gaze, but not reliably with alignment of the hands with the trunk midline.

*RT*. RT results are presented in Fig 2B. Analysis of RT revealed a significant crossing effect (main effect of Crossing Status, β = -0.10 (-0.13, -0.08), p < 0.001). The interaction of Crossing Status and *Eyes To Side* was not significant (β = 0.00 (-0.01, 0.01), p = 0.520). Instead, we observed a trend towards faster performance with crossed hands (β = -0.02 (-0.04, 0.00), p = 0.076; note, that the apparent inclusion of 0 in the confidence interval is due to rounding only), and significantly faster performance with uncrossed than crossed hands (β = -0.02 (-0.04, 0.00), p = 0.037) in *Eyes To Side*, suggesting that performance was slightly faster in the Eyes To Side condition overall.

There was a trend towards significance for the interaction of Crossing Status with *Eyes And Arms To Side* (β = -0.01 (-0.02, 0.00), p = 0.088). However, RT did not differ significantly between *All Straight* and *Eyes And Arms To Side* in uncrossed (β = -0.01 (-0.03, 0.01), p = 0.233) and crossed conditions (β = 0.00 (-0.02, 0.02), p = 0.787).

The direct comparison of *Eyes To Side* and *Eyes And Arms To Side* revealed neither a significant main effect of Eyes and Arms To Side (β = -0.02 (-0.03, 0.00), p = 0.132), nor an interaction of Eyes and Arms To Side and Crossing Status (β = 0.01 (0, 0.02), p = 0.259).

In sum, RT crossing effects did not consistently vary with any of the tested reference frame anchors.

## Experiment 2

### Participants

Twenty-three participants, 15 of them female, aged 20–37 years (mean: 26 years) took part in Experiment 2. Two of them were left-handed.

### Experimental design

**Fig 3:**
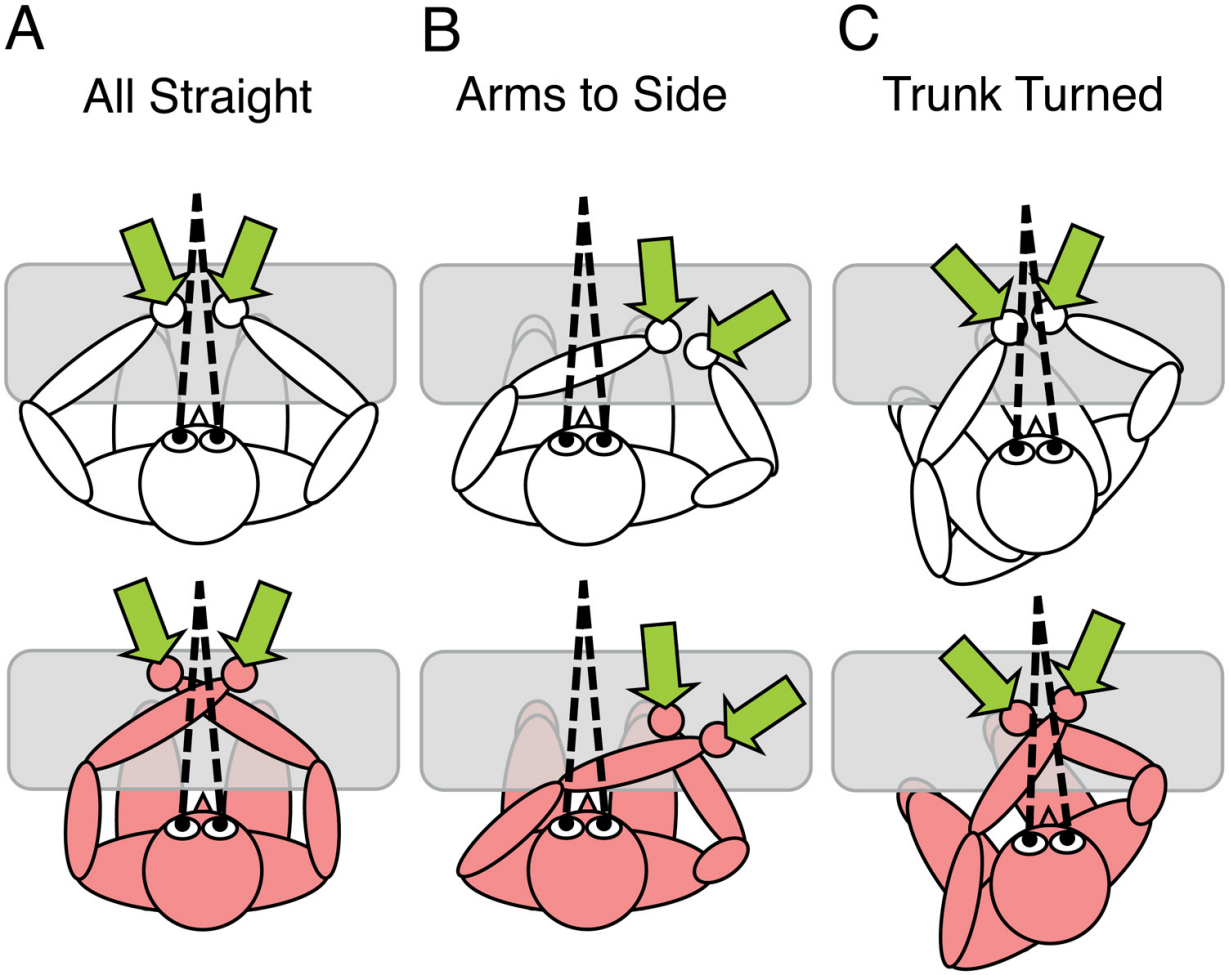
Experimental conditions of Experiment 2. (A-C) Posture conditions. Upper vs. lower row: uncrossed vs. crossed hand conditions. Dotted lines indicate fixation. Green arrows depict the locations of the two tactile stimuli for the temporal order judgment. See text for details.

Experiment 2 tested two aspects that arose from Experiment 1 (see Fig 3 for illustration of the conditions of Experiment 2). First, we sought to replicate the gaze-centered effect of Experiment 1. However, the reduction of the crossing effect in Experiment 1 had been observed in a condition in which the eyes were directed to the side, that is, eye position differed from the baseline condition. To exclude that the effects of gaze observed in Experiment 1 were due to the change of eye position rather than to the relative position of tactile stimulation to gaze, Experiment 2 included condition *Arms To Side* (Fig 3B). This condition differed from the baseline condition, *All Straight* (Fig 3A), only in that the arms were placed to the right, whereas eye, head, and arm position remained unchanged, so that the misalignment between arms and gaze was caused by a change in arm posture rather than by a change in eye position.

Second, Experiment 1 had suggested that trunk did not serve as anchor for an external reference frame for tactile TOJ. Yet, in Experiment 1 trunk was confounded with head position. Whereas the irrelevance of head position is consistent with the use of gaze as a combined eye/head position signal [38], the trunk has been suggested as a potential anchor for tactile localization [24], based on its relevance for visual spatial coding [39]. To scrutinize the null effect obtained in Experiment 1, therefore, in the third posture condition, *Trunk Turned*, the body was turned to the left underneath the head and eyes (Fig 3C), with the arms remaining fixed in their straight position. *Trunk Turned* was, therefore, identical to *All Straight*, except for trunk position. Thus, any TOJ effect observable in *Trunk Turned* relative to *All Straight* would indicate the use of a trunk-centered reference frame.

If tactile localization depended on a trunk-based reference frame, then the TOJ crossing effect should be reduced relative to *All Straight* both in *Arms To Side* and in *Trunk Turned* because the arms are misaligned with trunk midline in both of these conditions (Fig 3, crossing effect 3b < 3a, 3c < 3a). In contrast, if tactile localization depended exclusively on a gaze-centered reference frame, then condition *All Straight* and *Trunk Turned* should result in similar TOJ performance, but the TOJ crossing effect should be reduced for *Arms To Side*, because the arms are on one side relative to gaze only in the latter (Fig 3, crossing effect 3C = = 3A, 3B < 3A). Finally, if both trunk and gaze-centered reference frames were relevant for tactile localization, then condition *Arms To Side* should result in a reduced TOJ crossing effect relative to condition *All Straight*, and *Trunk Turned* should result in an additional reduction of the TOJ crossing effect relative to *Arms To Side* (Fig 3, crossing effect 3C < 3B < 3A).

In each posture condition, TOJ were administered with uncrossed and crossed hands, resulting in 6 combinations (3 posture conditions x uncrossed/crossed hands).

SOA between tactile stimuli, trial numbers, and experiment duration were as in Experiment 1.

### Materials

Setup and materials were largely identical to those of Experiment 1. However, participants directed gaze towards a fixation point attached to the wall in such a way that the eyes were always positioned straight in the head, and the eyes were not tracked. Participants placed their arms on a table in front of them. For the conditions in which the arms were positioned to the right of the trunk, participants' arms remained in the same position on the table, the trunk was turned by turning the seat of the chair by ~45°.

The experimenter continually monitored that participants remained in the required posture.

### Procedure

The experimental procedure was largely identical to that of Experiment 1. However, the inter-trial interval was set to 900–1200 ms, because, in contrast to Experiment 1, the start of a new trial was not gaze-contingent.

### Statistical analysis

Analysis strategy was analogous to that of Experiment 1. Posture condition *All Straight* served as base level for *Arms To Side* and *Trunk Turned*.

## Results

**Fig 4:**
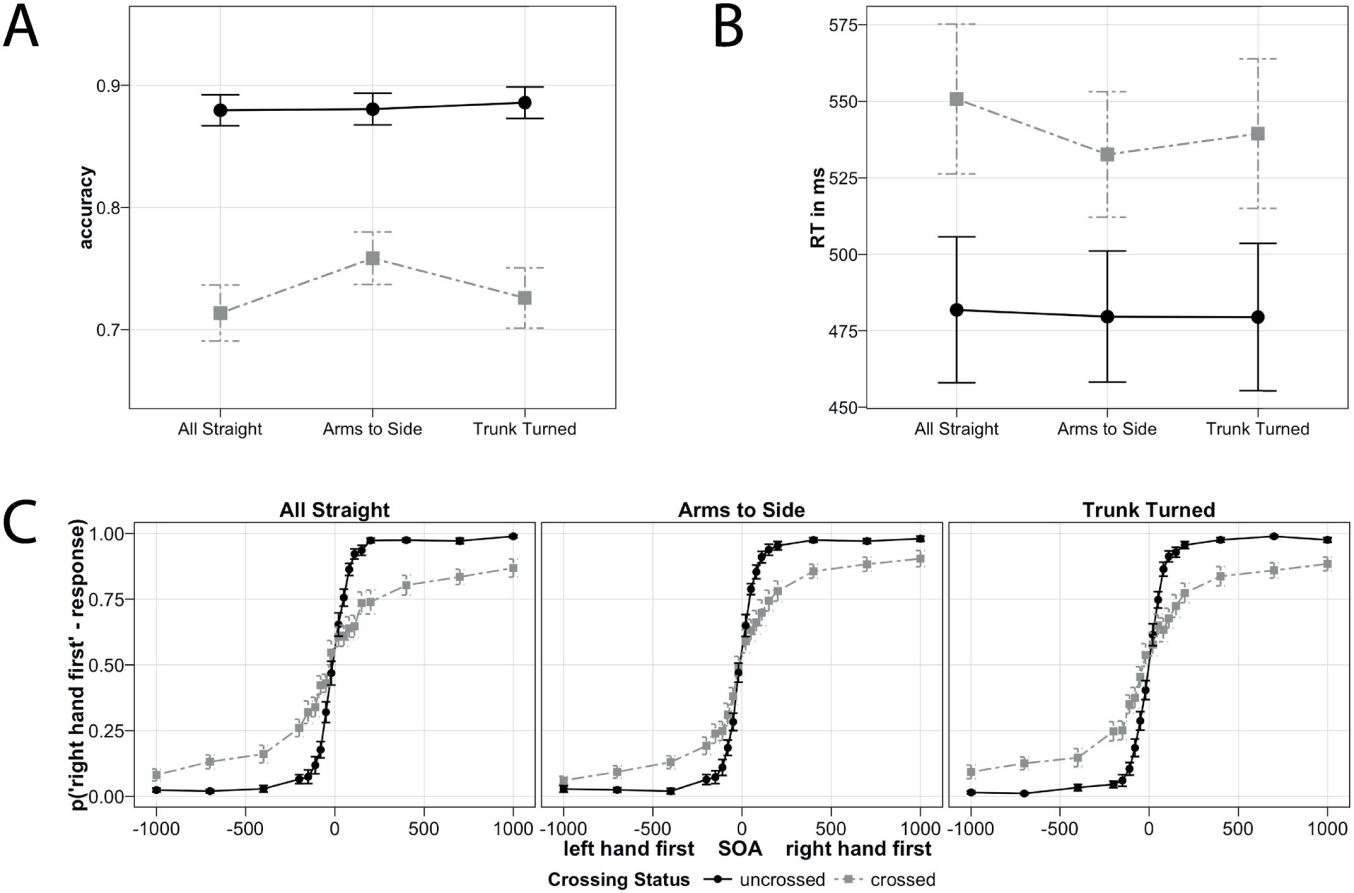
Results of Experiment 2. (A) Accuracy, depicted as the probability to report the correct stimulus. (B) Reaction time. (C) Accuracy recoded into proportions of ‘right hand first’ responses by SOA. (A,B,C) Black vs. grey color: uncrossed vs. crossed hand conditions. Error bars depict standard errors of the mean. Note, that y-axes do not start at 0.

Accuracy. Accuracy results are presented pooled across SOA in Fig 4A, and by SOA in Fig 4C. Crossing Status interacted with *Arms To Side* (β = -0.25 (-0.35, -0.14), p < 0.001). Thus, the hand crossing effect, that is, the difference in TOJ accuracy for uncrossed vs. crossed hands, was significantly reduced when the hands were misaligned with gaze and trunk. In the model that coded crossed hands of *All Straight* as baseline level, the simple effect of *Arms To Side* was significant as well (β = 0.26 (0.18, 0.34, p < 0.001), indicating higher TOJ accuracy in the *Trunk Turned* than in the *All Straight condition*. In contrast, the simple effect of *Arms To Side* was not significant in the model that was releveled to code uncrossed hands as baseline (β = 0.01 (-0.09, 0.11), p = 0.862). Thus, the *Arms To Side* interaction was driven by an improvement of TOJ accuracy in the crossed, but not in the uncrossed condition.

Crossing Status did not interact with *Trunk Turned* (β = -0.03, CI [-0.14, 0.07], p = 0.621). However, there was a trend towards a simple effect of *Trunk Turned* (β = 0.08, CI [0.01, 0.15], p = 0.076) in the crossed hands baseline model, suggesting that performance with crossed hands may have been affected by this condition. *Trunk Turned* did not significantly modulate TOJ performance in the model that coded uncrossed hands as baseline level (β = 0.05, CI [-0.05, 0.14], p = 0.417). Thus, if at all, then TOJ performance was affected in the crossed, but not in the uncrossed condition.

We releveled the GLMM with *Trunk Turned* as baseline condition to directly compare this condition with the *Arms To Side* condition. The interaction between Crossing Status and *Arms To Side* was significant (β = -0.21, CI [-0.32, -0.11], p < 0.001), indicating that the TOJ crossing effect differed between the *Arms to Side* and *Trunk Turned* Posture Conditions. The simple effect of *Trunk Turned* was significant for the crossed hand posture (β = 0.18, CI [0.10, 0.26], p < 0.001), but not for the uncrossed hand posture (β = -0.04, CI [-0.13, 0.06], p = 0.550). Thus, TOJ accuracy was improved in *Arms to Side* compared to *Trunk Turned* in the crossed, but not in the uncrossed condition.

In sum, accuracy was significantly affected when the arms were misaligned with gaze and trunk, but not when they were misaligned only with the trunk. This result implies relevance of a gaze but not of a trunk-centered reference frame.

*RT*. RT results are presented in Fig 4B. Crossing Status interacted with *Arms To Side* (β = 0.03 (0.02, 0.04), p < 0.001), and RT with crossed hands was slower when the hands were in separate than in the same hemifield (simple effect of *Arms To Side* in the crossed hands baseline model, β = -0.03 (-0.05, -0.01), p = 0.009). In contrast, RT was unaffected with uncrossed hands (uncrossed hands baseline model, simple effect of Posture Condition (3), β = 0, CI [-0.02, 0.02], p = 0.877).

Contrary to the accuracy results, the interaction between Crossing Status and *Trunk Turned* did reach significance for RT (β = 0.01, CI [0, 0.03], p = 0.046), although the confidence interval touched 0. Nevertheless, the simple effect of *Trunk Turned* was significant (β = -0.02, CI [-0.03, -0.01], p = 0.020) in the crossed hands baseline model, indicating that RT in the crossed condition was affected by a trunk-centered reference frame. In contrast, uncrossed performance was unaffected (releveled model, simple effect of *Trunk Turned*, β = -0.01, CI [-0.02, 0.01], p = 0.465).

The direct comparison of *Trunk Turned* and *Arms To Side* revealed a marginal interaction of Crossing Status with *Arms to Side* (β = -0.01, CI [0.00, 0.02], p = 0.054), but the simple effects that compared *Arms To Side* to *Trunk Turned* were not significant for uncrossed (β = 0.00, CI [-0.01, 0.02], p = 0.664) and crossed (β = -0.01, CI [-0.02, 0.01], p = 0.393) hands.

In sum, RT was significantly affected when the arms were misaligned with both gaze and trunk. In addition, there was a barely reliable modulation of RT when there was misalignment with the trunk alone. These results are suggestive of not only a gaze-centered, but also of a trunk-centered reference frame.

## Discussion

The present study had two aims. First, we wanted to bridge between direct localization tasks, such as pointing to tactile stimuli, and indirect localization paradigms, such as the tactile TOJ task, by testing whether a gaze-centered reference frame is relevant not only for direct, but also for indirect tasks. Second, we hypothesized that several external reference frames determine tactile localization; to this end, we tested whether a trunk centered external reference frame exhibits an influence on tactile spatial coding as well. The crossing effect in a tactile TOJ task, that is, the performance difference with uncrossed and crossed hands, served as a measure of the influence of gaze and trunk-centered spatial coding.

There were two main results. First, gaze had a significant influence on tactile localization, a finding that is well in line with the results obtained with other experimental paradigms [1–3,7,8,38,40].

Second, we observed, at most, weak evidence of trunk-centered coding. The relative position of touch to the trunk did not affect performance in Experiment 1. In contrast, Experiment 2 revealed an effect of relative trunk position in RT, but not in accuracy. Previous TOJ studies have often reported significant effects in accuracy that were not mirrored in RT [13,15], presumably because the TOJ task is typically unspeeded. The unusual, reverse result pattern in our experiment, thus, calls for cautious interpretation of these results. There is evidence for the existence of trunk-centered coding of visual target stimuli in the context of movement planning [41,42], as well as of vestibular information [43], in monkey posterior parietal cortex. Our results hint at the possibility that such trunk-centered coding may be relevant for tactile spatial coding as well, parallel to a more dominant gaze-centered reference frame.

The prevalence of a gaze-centered reference frame for touch localization is plausible for several reasons. First, tactile processing differs markedly in individuals who were born blind [18–21]. This point is maybe most strikingly illustrated by the fact that congenitally blind individuals do not exhibit the TOJ crossing effect upon which the current study was based, whereas individuals who lose sight later in life show a TOJ crossing effect just like sighted individuals [18]. Similarly, the position of the eyes remains relevant for proprioceptive spatial coding even after vision is lost due to disease or accident [44]. The relevance of a gaze-centered reference frame observed in the present study fits well with these links between the visual system and tactile spatial coding. Second, a gaze-centered reference frame is relevant not just in touch, but also in audition and proprioception [3,5,40,45], suggesting that it may serve as a code to integrate spatial signals across all modalities. Visual information is naturally bound to eye position because the visual receptors are arranged on the eye's retina. Using the reference frame that is directly related to one sensory system is sparser than using, say, a deduced, body-independent reference frame. Third, gaze appears to be central not only for sensory, but also for motor coding [46,47], possibly enabling efficient coding for eye-hand coordination [48,49]. Thus, a gaze-centered reference frame appears to connect perceptual and motor systems.

However, it is noteworthy that, although highly reliable, the effects of the gaze-centered reference frame were small in absolute terms; for instance, in Experiment 1, the change in accuracy attributable to the gaze-centered reference frame was from about 70 to 75%; in Experiment 2, effects were even smaller. Recall, that we argued in the Introduction that the misalignment of the hands with respect to a given reference frame should eliminate the conflict between anatomical and external reference frames. For instance, when both hands are placed in the right hemifield, then the right hand remains in the right hemifield even when the hands are crossed. Accordingly, a reference frame conflict between anatomical and external information should exist only for the left hand. Therefore, one might expect that the TOJ crossing effect would be reduced by half. In fact, in the case of binomial variables like correct/incorrect performance, changes in probability around 0.5 lead to larger changes of accumulated percent correct values than changes at higher probabilities, due to the non-linearity inherent in the log odds ratio [35]. Accordingly, an improvement in accuracy by about half would result in a change of even more than half of the percent correct value of the TOJ crossing effect. In contrast to this prediction, we observed large crossing effects, and accordingly a reduction of the effect by far less than half, with the off-center arm posture.

We speculate that a hand-centered reference frame is responsible for this observation [10,24]. In fact, when spatial conflict between anatomical and a hypothetical hand-based reference frames was reduced for just one hand (see Figure 5D in [10]), TOJ performance was ameliorated almost to the level of performance with uncrossed hands. Part of this effect may be caused by abandoning the physical crossing of the arms imminent in the postural manipulation used by those authors. Nevertheless, their result, together with the numerically small effects due to gaze position observed in the present study, suggests that TOJ crossing effects may depend, to a large degree, on reference frame that codes spatial information relative to the body parts involved in the tactile decision. For TOJ experiments with stimuli delivered to the hands, this type of coding then implies a hand-centered reference frame. The finding that the eye and trunk-related effects in the present study are far from accounting for the entire extent of the TOJ crossing effect, together with previous findings of the relevance of a hand-centered reference frame, suggest that a hand-centered reference frame is integrated as an additional external reference frame.

Thus, the present study suggests that not a single external reference frame determines spatial coding in touch, but that, instead, several external reference frames provide spatial information for tactile localization in parallel. Previously, TOJ crossing effects have been suggested to be a marker of the parallel use of an anatomical and an external reference frame [11,15,16,50], and previous results have hinted at the possibility that more than one reference frame is used to code the external location [13,14]. Electrophysiological work underlines the idea of the parallel use of different reference frames. For instance, the gaze-centered MEG alpha lateralization over parietal cortex during tactile motor planning is accompanied by anatomical lateralization of beta activity (15–30 Hz) in sensorimotor cortex [7,8]. Moreover, event-related potentials (ERPs) in the EEG evoked by tactile stimulation are modulated by both an external and an anatomical reference frame in the time range of 100–140 ms after tactile stimulation [51]. Crossing effects are evident in other tactile localization paradigms besides TOJ as well [50,52,53], and they correlate across tasks [50]. Therefore, the parallel availability of reference frames appears to be a general principle of tactile spatial coding, and the present study suggests that it extends to the parallel use of several external reference frames that are each anchored to a different body part. Presumably, the relative importance of each reference frame is set by context-specific reweighting. For instance, we have found that the size of hand crossing effects vary from task to task, but are correlated within individual participants [50]. Furthermore, TOJ performance is modulated by task load [29,54]. These results suggest that reference weights are assigned under consideration of the current task requirements under top-down control.

Some studies have suggested that the reference frames used for tactile localization may differ depending on the task context. At first sight, these studies seem to imply a switch, rather than the parallel use, of different reference frames. For instance, touch localization appears to rely on gaze-centered coding when a head, limb, or eye movement is made between tactile stimulation and the localization response; in contrast, body-centered coding was prevalent when no such intervening movement was made [3,40,55,56]. However, these results are also consistent with the view that multiple reference frames are available at any given time, and that the apparent switches of tactile reference frame coding reflect changes in the relative weights assigned to them [16,50,57].

In line with behavioral results in humans, monkey neurophysiology suggests the existence of multiple reference frames, such as eye [46], head [43,58], hand [59], body [42,43], and world-centered [42,43] ones, though these have partly been identified in different sensory modalities, such as vision, audition, and vestibulation. Different brain regions have been observed to exhibit specific preferences for the reference frames used to code spatial information [43], and the relevance of a given reference frame for the animal's behavioral output may depend on the task [60].

In conclusion, we observed a strong influence of gaze-centered coding on tactile localization, as assessed with the crossing effect in the tactile TOJ task. In addition, there was some, albeit weak, evidence for the concurrent use of a trunk-centered reference frame. The failure of these two reference frames to account for the total size of posture-induced effects suggests that a hand-centered reference frame, too, exhibits an influence on tactile spatial coding. The dependence of behavior on multiple spatial reference frames suggests that the brain estimates tactile location by integrating spatial information from multiple sources.
